# Bilateral Epiploic Appendagitis: A Rather Benign but Diagnostically Challenging Cause of Acute Abdominal Pain

**DOI:** 10.7759/cureus.7897

**Published:** 2020-04-30

**Authors:** Amman Yousaf, Soban Ahmad, Fariha Ghaffar, Sadia Sajid, Sundus Ikram

**Affiliations:** 1 Radiology, Hamad General Hospital, Doha, QAT; 2 Radiology, Services Institute of Medical Sciences, Lahore, PAK; 3 Emergency Medicine, Hamad Medical Corporation, Doha, QAT; 4 Internal Medicine, Allama Iqbal Medical College, Lahore, PAK; 5 Radiology, Hamad Medical Corporation, Doha, QAT; 6 General Surgery, Hamad Medical Corporation, Doha, QAT

**Keywords:** epiploic appendagitis, acute appendicitis, acute abdomen, conservative management, surgical exploration, case report, role of diagnostic imaging, literature review, acute diverticulitis, bilateral abdominal pain

## Abstract

Epiploic appendagitis (EA) is a rare and often misdiagnosed cause of acute abdominal pain. It is a benign and self-limited condition but mimics other underlying causes of acute abdominal pain like acute diverticulitis, acute appendicitis, acute cholecystitis, etc. Inaccurate diagnosis can lead to iatrogenic adverse outcomes. To the best of our knowledge, the present report represents the first case of bilateral EA involving both cecum and descending colon. The patient presented with symptoms of bilateral iliac fossa pain. Conservative management and close outpatient follow up resulted in a successful clinical outcome with no recurrence of symptoms. This article illustrates that clinicians and radiologists should include this etiology among differential diagnoses of patients presenting with acute abdominal pain, as it might prevent unnecessary hospitalizations, antibiotic therapy, and unwarranted surgical interventions.

## Introduction

Epiploic appendagitis (EA) is a rare yet benign cause of acute abdominal pain. It results from the torsion of the colonic appendages, resulting in thrombosis of the draining veins and causing aseptic inflammation of the affected epiploic appendages [1-2]. EA occurs predominantly in males in the fourth and fifth decade of life, with an approximate incidence of 8-9 cases per million individuals per year [3]. There have been some case reports in pediatric and geriatric age groups as well [4]. EA has been described more frequently among obese and overweight individuals. In a retrospective study by Nugent et al., it has been reported that patients with EA have 60% greater abdominal adipose volume and 117% increased visceral adipose area as compared to their control population [5]. Other possible causative factors are strenuous exercise routine, recent weight loss, and the presence of abdominal wall hernias [6].

Epiploic appendagitis usually presents with abdominal pain and perfectly mimics with other surgically managed conditions of serious nature. In the past, EA was also considered as a surgically treated pathology and often diagnosed during laparotomy [4, 7]. Advent of the latest radiologic imaging modalities, especially with the routine use of contrast-enhanced CT scan in patients with abdominal pain has resulted in increased recognition and diagnosis of EA, making it a significant entity for radiologists as well [8]. Prompt identification of pathognomonic radiological signs of EA can result in the avoidance of numerous unwanted surgical interventions, iatrogenic adverse patient outcomes, and futile dissipation of healthcare resources [9]. We describe here the case of a young patient who presented to the ED with bilateral lower abdominal pain secondary to bilateral EA and had a successful treatment with conservative management.

## Case presentation

A 26-year-old Asian male patient presented to the ED with lower abdominal pain for three days. Abdominal pain started in the left iliac fossa and later progressed to involve the right iliac fossa as well. The pain was dull, aching in character, nonradiating with no positional change, and was only partially relieved with intravenous paracetamol. The patient did not have any fever, nausea, or vomiting. His bowel habits were regular, and he denied dysuria, urinary frequency, and urgency. He never had similar pain episodes in the past. His past medical and surgical history was unremarkable.

On physical examination, he was slightly distressed due to abdominal pain. His initial vital signs examination showed blood pressure 124/85 mmHg, heart rate 92 beats per minute, respiratory rate 18 breaths per minute, temperature 37.4°C, and oxygen saturation of 98% on room air. The abdominal examination did not reveal any scar marks or abdominal wall hernias. There was significant tenderness in both right and left iliac fossae without any rebound tenderness or other signs of peritonism. Bowel sounds were audible. The per-rectal examination did not reveal any mass, fresh blood, or melena. The rest of the clinical review was unremarkable.

**Figure 1:**
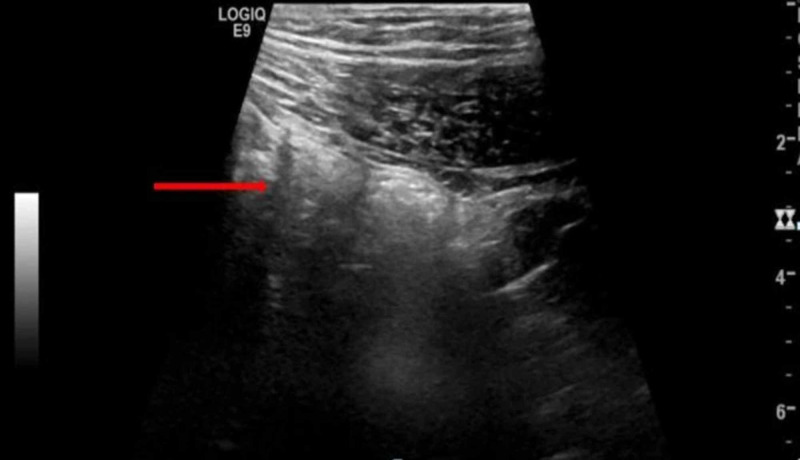
Ultrasound abdomen. A selected gray-scale ultrasound image taken with a curvilinear low frequency probe depicting the right iliac fossa. The overlying echogenic gassy bowel loops (red arrow) are making the visualization of the underlying structures difficult. There is no identifiable cylindrical structure (appendix).

Laboratory studies revealed mild leukocytosis (11.3 x 109/L), hemoglobin (16.3 g/dL), and thrombocytes (220 x 109/L). C-reactive protein (CRP) level was also mildly elevated (7.8 mg/dL). Liver function tests revealed slightly elevated alanine aminotransferase (ALT) levels at 55 IU/L. Urinalysis was unremarkable for leukocytes and nitrite. Serum amylase, lipase, sodium, and potassium were within normal limits. His initial abdominal ultrasound examination demonstrated probe tenderness in the right iliac fossa. However, appendix could not be identified due to overlying gassy bowel loops (Figure 1).

**Figure 2:**
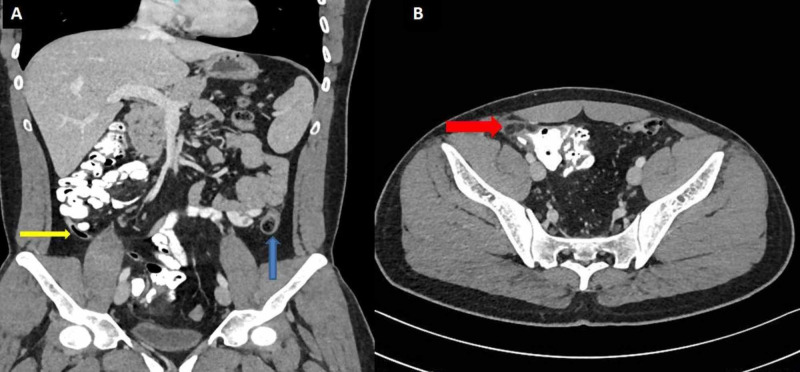
Contrast-enhanced CT abdomen and pelvis. A sagittal section of CT abdomen and pelvis shows normal appendix (yellow arrow) and a fat density oval-shaped lesion (blue arrow) anterior to the descending colon with perilesional fat stranding and a minimal amount of free fluid (A). An axial section of the CT abdomen and pelvis shows a second fat density structure (red arrow) anterior to the cecum with peripheral fat standing, free fluid, and adjacent cecal wall thickening (B).

Due to clinical suspicion of acute appendicitis and acute diverticulitis, a contrast-enhanced CT abdomen was performed, which showed a normal-sized appendix in the right iliac fossa, with no peri-appendicular inflammatory changes (Figure 2A). An oval fat density mass attached to the anterior cecum, measuring 2.4 cm in maximum diameter with surrounding fat stranding and a thin rim of free fluid was noted (Figure 2B). Another similar oval fat density mass was present anterior to the descending colon, measuring 2 cm in diameter with surrounding inflammatory changes (Figure 2A).

Radiological features of both these lesions confirmed the diagnosis of bilateral EA. Based on the clinical features and pathognomonic radiological evidence of EA, our patient received symptomatic treatment with intravenous ketorolac in the ED, which successfully alleviated his symptoms. The patient was reassured regarding the benign nature of his condition and was discharged home on oral ibuprofen (NSAIDs) from the ED. At a one-week follow up, the patient did not report the recurrence of his symptoms. The patient continues to do well to date.

## Discussion

Epiploic appendages anatomy was first described by Suresh Kumar et al. in 2019 [10]. They consist of pedunculated fat-containing outpouchings attached on the anti-mesenteric surface of the colonic wall [4]. There are approximately 50-100 peritoneal membrane-covered epiploic appendages in the entire adult colon with abundance over transverse and sigmoid colon [11]. Their vascular supply comprises one or two arteries and one draining vein, and their length varies between 0.5 and 5 cm. Epiploic appendages develop during the fifth-sixth months of intrauterine life, and their size remains small during childhood. They increase in size during adulthood, especially in the obese population due to abundant visceral fat content [6, 10]. The exact functional role of epiploic appendages is not well understood. However, a hypothesis suggests that these appendages act as cushions providing mechanical protection for bowel loops, and as a source of blood supply for colonic walls during peristalsis. Other theories highlight their role in fat storage and immune function [12].

Epiploic appendages, because of their narrow peduncles, are prone to undergo torsion with consequent vascular supply impairment, initially affecting the venous return resulting in ischemia of appendages. Spontaneous thrombosis of the draining vein can also result in a similar outcome [4, 13]. Ischemia of epiploic appendages leads to wall edema, necrosis, and aseptic inflammation of the affected appendages that are called EA. This inflammatory process eventually leads to absorption of the inflamed appendages by the peritoneal cavity and can be identified as peritoneal loose bodies, also known as ‘peritoneal mice’ when visualized on radiological imaging or intra-operatively [14]. Other possible causes of EA reported in the literature are hernia incarceration and intestinal obstruction [10].

The clinical presentation of EA is vague and similar to other conditions causing acute abdominal pain such as acute diverticulitis, acute appendicitis, acute cholecystitis, ovarian torsion, and ectopic pregnancy in females. Patients usually present with sudden onset of localized left or right iliac fossa pain that worsens with cough and local compression. The most common presenting complaints in patients with EA are left lower quadrant abdominal pain and right lower quadrant abdominal pain, mimicking acute diverticulitis and acute appendicitis, respectively [10]. It is rare to have bilateral iliac fossa pain, as described by our patient, and no similar case has been reported in the literature thus far. Abdominal examination is usually unremarkable apart from localized tenderness and rarely rebound tenderness [15]. Laboratory investigations in patients with EA are generally within normal limits, and if present, are nonspecific, including mild leukocytosis and raised inflammatory serum markers just like our patient.

Lack of specific clinical and laboratory findings, as well as the lack of awareness of EA among clinicians, makes it a diagnostic challenge. It is practically impossible to diagnose EA without radiological imaging. Before the era of CT scan, EA was a diagnosis of exclusion and confirmed during laparoscopy or laparotomy with direct visualization of inflamed appendages [4, 10].

With the widespread use of CT in the evaluation of patients with abdominal pain and increasing awareness of this diagnosis among radiologists, reported incidence of EA is increasing. A CT scan can easily recognize EA in the presence of its typical pathognomonic findings. The hallmark radiologic finding in EA is the presence of a fat density, round to ovoid shape mass anterior to colon measuring between 1.5 and 3.5 cm in diameter, with surrounding fat stranding and free fluid (100%). Nugent et al. reported the frequencies of common CT findings in EA as an ovoid mass with hyper-attenuation ring (100%), central hyperdense dot sign (79%), peritoneal thickening (76%), or bowel wall thickening (47%) [5]. 

Epiploic appendagitis is a self-limited disease. Once diagnosed with imaging, most patients can be managed conservatively with or without using a short course of oral anti-inflammatory drugs (most commonly NSAIDs). The main goal of prescribing anti-inflammatory medication is to provide analgesia, as their role in modifying disease course is not proven [4, 10]. Most patients do not require antibiotic therapy, hospitalization, or invasive surgical intervention and can be safely discharged home from the ED [9].

## Conclusions

Epiploic appendagitis is a rare but diagnostically challenging cause of the acute abdomen, especially when the patients have bilateral abdominal pain, and clinicians have a low index of suspicion. This article emphasizes on the awareness of this clinical entity amongst clinicians and the identification of typical CT findings by radiologists that can reduce unnecessary antibiotic use, unwanted surgical consults, and unwarranted surgical interventions. Most importantly, it would reduce patient costs and morbidity. This article also highlights a further need for research work to explore the exact pathophysiology and possible etiologies of EA.
